# Shifting Perspective in Influenza Vaccines Efficacy: How Risk Difference Shows an Alternative View of the Comparative Efficacy Profile of Newer and Enhanced Influenza Vaccines Compared to Standard, Egg-Based Vaccines

**DOI:** 10.3390/vaccines14010108

**Published:** 2026-01-22

**Authors:** Laura Colombo, Abraham Palache, Sanjay Hadigal

**Affiliations:** 1 Global Medical Affairs, Viatris, Viale Piero e Alberto Pirelli, 10, 20126 Milan, Italy; 2 FluPal Consultancy, Bourgondischelaan 33, 1181 DC Amstelveen, The Netherlands; 3 Global Medical Affairs, Viatris, Prestige Tech Park, Platina-3, Kadubesanahalli, Bengaluru 560103, India

**Keywords:** influenza vaccines, vaccine efficacy, risk difference (RD), number needed to vaccinate (NNV), public health impact

## Abstract

**Highlights:**

**What are the main findings?**

- Novel influenza vaccines—including HD-IIV, rIV, cIV, and aIV—show superior relative vaccine efficacy (rVE) compared with standard-dose inactivated influenza vaccines (SD-IIV).
- However, when examined through the risk difference (ΔRD) and the number needed to vaccinate (ΔNNV), the absolute benefit at the population level is modest, with fewer than 10 additional cases prevented per 1000 vaccinations.

**What are the implications of the main findings?**

- Although newer influenza vaccines offer improved relative efficacy, the modest absolute benefit highlights that standard-dose influenza vaccines remain highly relevant and valuable for public health.
- This suggests that while enhanced vaccines provide incremental improvements, broadening immunization continues to be an important and effective strategy, irrespective of vaccine type, especially when considering cost, availability, and overall impact at the population level.

**Abstract:**

Annual influenza vaccination remains critical for mitigating severe illness and reducing healthcare strain, particularly among high-risk populations. Despite advancements in vaccine platforms, the comparative efficacy of novel vaccines—such as high-dose (HD-IIV), recombinant (rIV), cell-based (cIV), and adjuvanted (aIV) influenza vaccines—versus standard-dose non-adjuvanted (SD-IIV) vaccines remains a public health concern. Traditional Relative Vaccine Efficacy (rVE) metrics, though robust, may overestimate population-level benefits. This short communication explores alternative comparative efficacy measures: risk difference (ΔRD) and number needed to vaccinate (ΔNNV). Analysis of data derived from randomized controlled trials (RCTs), or robust pragmatic trials, shows that while rVE values for newer vaccines often indicate superior efficacy, ΔRD and ΔNNV highlight the limits in incremental protection at the population level, with ΔRD generally below 10 cases per 1000 vaccinated. These findings underline the sustained relevance of SD-IIV in immunization programs and emphasize the need for broader vaccine coverage to highlight the benefits of vaccination and enhance population health outcomes.

## 1. Introduction

Influenza remains a global health threat, causing a significant socioeconomic burden. It strains healthcare systems and leads to reduced productivity. This underscores the urgent need for effective prevention and management strategies [1].

Annual vaccination is the most effective way to prevent severe disease, reduce epidemic impact, and alleviate strain on healthcare systems [2]. It serves dual purposes: direct protection of vaccinated individuals and indirect protection through reduced viral circulation. Influenza vaccines with an optimal (absolute) efficacy profile can better protect people from the viral infection and its consequences; in addition, when vaccine coverage is sufficiently high, herd immunity can protect individuals who cannot be vaccinated. Vaccination is especially crucial for high-risk groups. Despite this, influenza vaccination coverage remains suboptimal in most countries, limiting the full potential of immunization programs [2].

**Figure 1:**
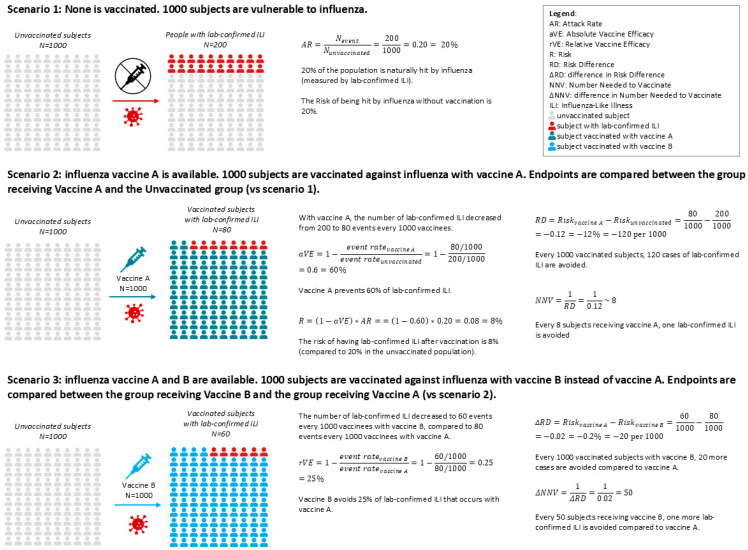
Example of natural incidence of influenza in an unvaccinated population (Scenario 1), and how it changes when the population is vaccinated with two different vaccines (Scenarios 2 and 3). Scenario 1: Natural incidence of influenza. Scenario 2: The Absolute Vaccine Efficacy aVE represents how well a vaccine can prevent the disease. Scenario 3: If the placebo arm is absent in an RCT comparing vaccine A with vaccine B, it is not possible to calculate the AR and the aVE for each vaccine and then compare the two to calculate the relative efficacy. Regardless, estimates of relative values are possible. Vaccination could be more impactful in the case of higher aVE, or if the disease has a higher attack rate (more cases to prevent).

Traditionally, the vaccine efficacy/effectiveness (VE) measures how well a vaccine prevents specific flu-related outcomes (see Figure 1), but could depend on various vaccine-independent factors such as vaccine type, influenza season, geography, matching status, and subject subgroup [3]. To enhance protection, newer vaccines with higher antigen content have been developed, the high-dose influenza vaccine (HD-IIV, 60 µg HA per strain) and the recombinant influenza vaccine (rIV, 45 µg HA per strain), which are more immunogenic compared to standard-dose vaccines (SD-IIV, 15 µg HA per strain) and could provide better protection [4,5,6]. Additionally, rIV and cell-based influenza vaccines (cIV) avoid egg-based manufacturing, allowing for faster production, avoiding the risk of egg shortages and of potential egg-adaptive mutations [7]. Further, adjuvants like MF59 can be added to the vaccine formulation to increase the immune response in the recipients [8].

Beyond VE, population-level metrics such as risk difference (RD) and number needed to vaccinate (NNV) offer valuable insights for public health planning: the RD measures the proportion of patients who avoid the infection-related outcome because of being vaccinated [9], while the NNV is the number of people who need to be vaccinated to prevent one outcome event, taking into account both the efficacy of the vaccine and the incidence of the disease [10]. These metrics quantify the absolute benefit of vaccination and help policymakers assess the real-world impact of vaccine strategies. When comparing newer vaccines to SD-IIV, these measures become relative (relative vaccine efficacy/effectiveness [rVE], difference in risk difference [ΔRD], and difference in number needed to vaccinate [ΔNNV]), providing a more nuanced understanding of incremental benefits (Figure 1).

It is well established that a high rVE value obtained in clinical trials, although describing a better performance of one vaccine over the other, may not always translate into substantially greater absolute benefits at the population level, and does not necessarily reflect the actual extent of added public health benefit over the standard of care. The difference in ΔRD and ΔNNV could be more relevant from a societal perspective.

This study aims to compare the public health benefits of newer vaccines and SD-IIV by estimating and comparing the respective ΔRD and ΔNNV, specifically focusing on clinical efficacy outcomes measured in randomized controlled trials (RCTs).

## 2. Materials and Methods

Several authors presented comprehensive systematic reviews and meta-analyses based on the extensive available literature on newer IV formulations (HD−IIV, cIV, rIV, aIV) [11]. We screened the reference lists of these reviews for articles that contain eligible study data on HD-IIV, cIV, or rIV (umbrella retrieval approach) [12] and focused our search on comparative RCTs against SD-IIV assessing lab-confirmed influenza-like illness (ILI).

We made an exception in the case of the adjuvanted vaccine (aIV): for this formulation, there is a lack of comparative RCTs against SD-IIV assessing lab-confirmed ILI, as highlighted in a previous systematic review [11], and only a few observational studies provided effectiveness estimates against lab-confirmed influenza, with highly heterogeneous results. For this review, a pragmatic trial [13] comparing adjuvanted vaccines with non-adjuvanted influenza vaccines against flu-related hospitalizations was included.

For each vaccine type, one representative study was selected to calculate rVE, ΔRD, and ΔNNV.

Calculations were based on reported event rates in the selected studies. ΔRD was defined as the difference in absolute risk between newer vaccines and SD-IIV, while ΔNNV was calculated as the inverse of ΔRD. These metrics were used to assess the incremental population-level benefit of newer vaccines over SD-IIV. Confidence intervals were included to evaluate statistical significance, and comparisons crossing zero were noted as not statistically significant (Table 1).

## 3. Results

### 3.1. Population Benefits Comparison of HD-IIV vs. SD-IIV

The DiazGranados 2014 [4] study evaluated HD-IIV in elderly populations, reporting a statistically significant rVE of 24% (95% CI [9.7, 36]) against lab-confirmed ILI (Table 2).

Based on the data reported in the study, the ΔRD_HD|SD_ against any lab-confirmed protocol-defined ILI was statistically significant at 4.6 per 1000 (95% CI [1.8, 7.4]) vaccinated individuals; the statistically significant ΔNNV_HD|SD_ was 219 (95% CI [136, 565]).

There is limited available data about the efficacy profile stratified by virus subtype or matching status. Subgroup analyses showed ΔRD values of 2.3 (95% CI [−0.064, 4.6]) for matched B strains and 4.4 (95% CI [−0.2, 9.1]) (unmatched A-H3N2 strains) per 1000, though these were not statistically significant. Notably, the rVE was higher for matched strains at 31% (95% CI [−1.0, 53]) than for unmatched at 16% (95% CI [−1, 30]), and both were not statistically significant, highlighting the complexity of interpreting efficacy across subtypes. The ΔNNV_HD|SD_ values for matched B strains and unmatched A-H3N2 strains were 436 (95% CI [22, ∞]) and 222 (95% CI [11, ∞]), respectively, with both being statistically significant.

### 3.2. Population Benefits Comparison of rIV vs. SD-IIV

The Dunkle 2017 paper compared the efficacy of rIV with SD-IIV in older adults (above 50 years of age) during the 2014–2015 influenza season [6]. The study showed that an rVE_rIV|SD_ of 30% (95% CI [10, 47]) overall and an rVE_rIV|SD_ of 36% (95% CI [14, 53]) during a season with an antigenic mismatch were both statistically significant. Antigenically matched rVE was 4.2% (95% CI [−72, 46]), which was not statistically significant.

The ΔRD_rIV|SD_ against any lab-confirmed protocol-defined ILI was statistically significant at 9.8 per 1000 (95% CI [2.9, 17]) persons. The corresponding ΔNNV_rIV|SD_ was 102 (95% CI [59, 345]), which was statistically significant. In case of antigenic mismatch, ΔRD_rIV|SD_ is 9.1 per 1000 persons (95% CI [3.0, 1.5]), and the corresponding ΔNNV_rIV|SD_ is 110 (95% CI [66, 332]), with both statistically significant. The ΔRD_rIV|SD_ and ΔNNV_rIV|SD_ were 0.23 per 1000 (95% CI [−0.29, 3.3]) and 4256 (95% CI [303, ∞]), where neither were statistically significant (Table 2).

These results suggest that rIV may offer greater benefit in seasons with poor antigenic match, though the absolute gains remain modest.

### 3.3. Population Benefits Comparison of cIV vs. SD-IIV

The Frey 2010 study assessed cIV in adults under 50 and reported a not statistically significant rVE of 17% (95% CI [−24, 45]) against lab-confirmed ILI [14]. The ΔRD_cIV|SD_ was 2.3 per 1000 persons (95% CI [−2.7, 7.4]), not statistically significant; and ΔNNV_cIV|SD_ was statistically significant at 426 (95% CI [13, ∞]). In matched H1N1 seasons, rVE reached 40% (95% CI [−84, 80]), not statistically significant, with the respective ΔRD_cIV|SD_ being not statistically significant at 0.87 per 1000 persons (95% CI [−0.010, 2.8]) and statistically significant ΔNNV_cIV|SD_ being 1143 (95% CI [358, ∞]). For mismatched B strains, rVE was −4% (95% CI [−74, 39]) and not statistically significant, and the ΔRD_cIV|SD_ was not statistically significant at −0.26 per 1000 (95% CI [−4.2, 3.7]). The ΔNNV_cIV|SD_ was statistically significant and estimated to be less than 0. These findings highlight the limited incremental benefit of cIV in younger populations (Table 2).

### 3.4. Population Benefits Comparison of aIV vs. Non-Adjuvanted SD-IIV

The McConeghy 2021 study compared the immunogenicity and effectiveness of the aIV versus the non-adjuvanted SD-IIV in preventing hospitalizations in nursing homes among older adults (≥65 years) [13]. The study reported a statistically significant rVE of 21% (95% CI [6.7, 33]) against pneumonia and influenza-related hospitalizations. The ΔRD_aIV|SD_ was 2.6 per 1000 patients (95% CI [0.08, 4.4]), while the corresponding ΔNNV_aIV|SD_ was 383 (95% CI [225, 1282]), with both being statistically significant.

## 4. Discussion

The results of our analysis demonstrate the population-level impact of influenza vaccination using available vaccine formulations. SD-IIV provide a foundational level of protection, while newer and enhanced vaccines—such as HD-IIV, rIV, cIV, and aIV—offer modest incremental benefits when evaluated through ΔRD_new|SD_ and ΔNNV_new|SD_ metrics.

The two measures included, ΔRD_new|SD_ and ΔNNV_new|SD_, provided a different perspective on the added population protection related to uptake of newer vaccines. While ΔRD_new|SD_ per 1000 shows how many more people are protected by the new vaccines if 1000 persons receive the new vaccines instead of the SD-IIV, the ΔNNV_new|SD_ is the number of persons who should receive the newer vaccine (besides the ones receiving the SD-IIV) to prevent one extra outcome (over the cases already prevented by the SD-IIV).

**Figure 2:**
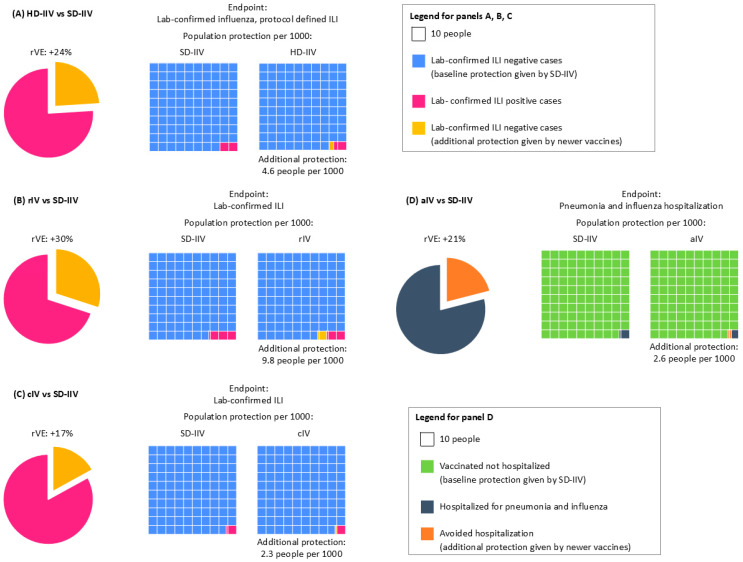
Relative Vaccine Efficacy and impact of vaccinating people with (**A**) HD-IIV, (**B**) rIV, (**C**) cIV, or (**D**) aIV compared with SD-IIV. Panels A, B, and C show rVE against lab-confirmed ILI, as extrapolated from RCTs Diaz Granados 2014 [4], Dunkle 2017 [6], and Frey 2010 [14]. Panel D shows rVE against pneumonia and influenza hospitalization, as extrapolated from the pragmatic trial McConeghy 2021 [13]. The four cases show the following: pie chart on the left, the rVE or each vaccine compared to SD-IIV (in yellow), and on the right, the outcome incidence rate with the two vaccines (pink boxes for A, B, and C; gray boxes for D). The yellow boxes in A–D highlight the additional number of cases avoided with the use of newer vaccines compared to the baseline cases avoided by SD-IIV.

While rVE values for the four newer vaccines often appear favorable, they do not necessarily translate into substantial public health gains. For example, ΔRD values generally remain below 10 cases per 1000 vaccinated individuals. Indeed, it is well known that when all the vaccines are effective and rVE is high, the ΔRD could remain small if the disease incidence is relatively low. The calculated NNV is high, indicating that a large number of individuals must be vaccinated to prevent a single additional case [15,16]. Conversely, the smaller the ΔNNV, the more impactful the newer vaccine is compared to the SD-IIV. Overall, a favorable rVE is not indicative of significantly better population protection conferred by the new vaccines compared to the SD-IIV, while the ΔRD and the ΔNNV indicate that SD-IIV still has good population protection rates, and that newer vaccines can provide modest benefits. This underscores the importance of interpreting efficacy data through a population lens, especially when informing public health strategies (Figure 2).

From a policymaker’s perspective, these findings suggest that the modest protection gains of newer vaccines may not justify widespread replacement of SD-IIV in national immunization programs, particularly given cost considerations or logistical constraints.

For instance, people vaccinated with HD-IIV are at lower risk of developing lab-confirmed ILI than those receiving SD-IIV (rVE 24%), but the incremental protection provided by HD-IIV, as highlighted by small ΔRD values, is modest (Figure 2A). Achieving additional benefits on a societal level requires a large number of vaccinees to materialize. Further, a recent publication has also reported that the HD vaccine showed limited incremental benefit in reducing hospitalizations compared to the SD vaccine, even in high-risk populations [17]. Similarly, rIV and cIV demonstrate improved rVE in certain scenarios, such as antigenic mismatch seasons, but their ΔRD values remain modest, and ΔNNV values are often prohibitively high (Figure 2B,C). rIV seemed to offer high protection against lab-confirmed ILI compared to SD-IIV (rVE 30%), which can be higher (rVE 36%) during seasons with an antigenic mismatch (Figure 2B). Also for rIV, when considering population benefits, the data available so far did not provide evidence of a better performance of rIV against SD-IIV, as the ΔRD remains below 10 cases per 1000 vaccinated persons (Figure 2B).

The efficacy profile of cIV suffers similar issues: while rVE against lab-confirmed ILI is 17%, reaching up to 40% in the case of the H1N1 predominantly matched season, the benefits at the population level are less evident, with only 2.3 additional people better protected per 1000 persons (down to 0.87 in case of H1N1 predominantly matched season) (Figure 2C).

aIV showed an efficacy of 17% against pneumonia and influenza-related hospitalization over non-adjuvanted SD-IIV; however, only 2.6 people per 1000 were better protected (Figure 2D).

A recent study showed that rIV performs significantly better against influenza infection (PCR confirmed), but there is no significance for hospitalization endpoints [18]. Further, it is associated with a larger NNV, as around 3000 individuals must be vaccinated with the recombinant vaccine to prevent one extra case of lab-confirmed influenza compared to the SD-IIV in certain age groups [19].

One limitation of our study is that we considered only one influenza-related endpoint/outcome for practical reasons. Meta-analyses have shown that HD-IIV may reduce all-cause hospitalizations by approximately 11% compared with SD-IIV [20], and influenza vaccination overall is associated with significant reductions in cardiovascular mortality [21]. These findings underscore that influenza vaccine impact extends beyond influenza case prevention and includes secondary benefits that are clinically and economically relevant. Nevertheless, given the epidemiological characteristics of influenza, the magnitude of benefit depends primarily on population coverage rather than vaccine type. We intend to emphasize that rVE, ΔRD, and ΔNNV should be interpreted in the context of their calculation, not as standalone values. Broader endpoints such as all-cause hospitalizations or mortality are clinically relevant, but estimating their population-level impact would require assumptions that expose them to high risks of bias. For this reason, we focused on consistently reported endpoints from RCTs and pragmatic trials, while acknowledging that the ultimate goal of vaccination is to reduce severe outcomes and improve public health.

While our analysis focused on comparative efficacy metrics, vaccine platform characteristics such as production timelines and avoidance of adaptive mutations could be relevant for public health planning in the case of pandemic scenarios, where rapid scale-up or improved antigenic fidelity are critical for disease control.

Importantly, the RD and NNV metrics focus on direct protection and do not account for indirect effects such as reduced transmission and herd immunity. As highlighted in previous studies, the full impact of vaccination is nonlinear and extends beyond individual-level efficacy [22]. Therefore, while newer vaccines may offer advantages in specific subpopulations, broad vaccine coverage remains the most effective strategy for maximizing public health outcomes [23,24].

## 5. Conclusions

This comparative analysis confirms that while newer and enhanced influenza vaccines offer incremental benefits, SD-IIV continues to play a critical role in influenza control at the population level. This overall conclusion aligns with the assessment of the WHO, which advocates the use of standard vaccines that are broader in coverage, more widely available, and more affordable than newer vaccines. For policymakers, the decision to adopt newer vaccines should be guided not only by rVE but also by ΔRD, ΔNNV, and broader public health considerations. Given the modest population-level impact (small reduction in ΔRD, large estimate for ΔNNV) and higher costs associated with newer vaccines, maintaining widespread access to SD-IIV in national immunization programs targeting at-risk populations is a pragmatic and effective approach. Newer and enhanced vaccines will continue having a role in providing improved protection for at-risk groups with specific risk factors, and to guarantee supply chain resilience and manufacturing diversification, which is important for long-term planning but beyond the scope of this analysis. Policymakers must weigh these implications when designing immunization strategies.

Furthermore, indirect benefits of vaccination—such as reduced transmission and enhanced herd protection—are not captured by rVE, ΔRD, or ΔNNV metrics but are essential to achieving long-term public health goals.

Beyond direct influenza control, vaccination irrespective of type contributes to reducing severe outcomes, including hospitalizations and cardiovascular mortality, as highlighted by recent consensus recommendations [25]. These secondary benefits reinforce the importance of broad coverage as the primary driver of public health impact.

Increasing coverage is a key public health priority and aligns with global health policy goals. Altogether, this evidence supports the need for further efforts to increase vaccination uptake with the currently available vaccines, rather than focusing on selective uptake of newer formulations in specific and small subgroups. Our findings are fully in line with the Vaccines against influenza: WHO position paper—May 2022 [1].

In summary, a population-focused strategy that emphasizes coverage and equity in vaccine access will yield the greatest public health benefit, supporting the continued use of SD-IIV as a cornerstone of influenza prevention.

## Figures and Tables

**Table 1:** Definitions and formulas.

Parameter	Definition	Formula
Attack Rate (AR)	The attack rate AR describes how many unvaccinated subjects are naturally hit by influenza (disease measures with a specific outcome).	$AR=\frac{N_{outcome\;event}}{N_{unvaccinated}}$
Absolute Vaccine Efficacy (aVE)	The Absolute Vaccine Efficacy aVE represents how effective the vaccine is in preventing a specific outcome.	$aVE=1-\frac{{event\;rate}_{vaccine\;A}}{{event\;rate}_{unvaccinated}}$ $aVE=1-\frac{\frac{N_{outcome\;event\;in\;vaccinated\;with\;A}}{N_{vaccinated\;with\;A}}}{\frac{N_{outcome\;event\;in\;unvaccinated\;}}{N_{unvaccinated}}}$
Risk (R)	The risk expresses the risk of having the disease (measured with a specific outcome) in the vaccinated population compared to the unvaccinated population.	$R=\left(1-aVE\right)*AR$
Risk Difference (RD)	The risk difference RD expresses how much the risk is diminished in the vaccinated population compared with the unvaccinated population.	$RD={Risk}_{vaccine\;A}-{Risk}_{unvaccinated}$ $RD=\frac{N_{outcome\;event\;in\;vaccinated\;with\;A}}{N_{vaccinated\;with\;A}}\;\;\;\;\;\;\;\;\;\;\;\;\;\;\;\;\;\;\;\;\;\;\;\;\;\;\;\;-\frac{N_{outcome\;event\;in\;unvaccinated\;}}{N_{unvaccinated}}$
Number Needed to Vaccinate (NNV)	The number needed to vaccinate NNV represents the number of people to be vaccinated to avoid one specific outcome.	$NNV=\frac{1}{RD}$
Relative Vaccine Efficacy (rVE)	The Relative Vaccine Efficacy rVE represents the proportion of outcomes avoided by vaccine B compared to vaccine A.	$rVE=1-\frac{{event\;rate}_{vaccine\;B}}{{event\;rate}_{vaccine\;A}}$ $rVE=1-\frac{\frac{N_{outcome\;event\;in\;vaccinated\;with\;B}}{N_{vaccinated\;with\;B}}}{\frac{N_{outcome\;event\;in\;vaccinated\;with\;A\;}}{N_{vaccinated\;with\;A}}}$
Difference in Risk Difference (ΔRD)	The difference in risk difference ΔRD expresses how much further the risk is diminished with vaccine B compared to vaccine A.	$\Delta RD={Risk}_{vaccine\;A}-{Risk}_{vaccine\;B}$ $RD=\frac{N_{outcome\;event\;in\;vaccinated\;with\;A}}{N_{vaccinated\;with\;A}}\;\;\;\;\;\;\;\;\;\;\;\;\;\;\;\;\;\;\;\;\;\;\;\;\;\;\;\;-\frac{N_{outcome\;event\;in\;vaccinated\;with\;B}}{N_{vaccinated\;with\;B}}$
Difference in Number Needed to Vaccinate (ΔNNV)	The difference in number needed to vaccinate ΔNNV represents the number of subjects to be vaccinated with vaccine B to avoid one additional outcome compared to vaccine A.	$\Delta NNV=\frac{1}{\Delta RD}$

**Table 2:** Vaccine-specific event rates, extrapolated from RCTs Diaz Granados 2014 [4], Dunkle 2017 [6], and Frey 2010 [14], and respective Relative Vaccine Efficacy (rVE), risk difference (ΔRD), and number needed to vaccinate (ΔNNV). A) HD-IIV vs. SD-IIV; B) rIV vs. SD-IIV; C) cIV vs. SD-IIV; D) aIV vs. SD-IIV.

**A**	**SD-IIV**			**HD-IIV**						
**Clinical Endpoint**	**Vaccinated**	**Positive**	**Event Rate**	**Vaccinated**	**Positive**	**Event Rate**	**rVE** **(95%CI)**	**ΔRD % (95%CI)**	**ΔRD per 1000**	**ΔNNV** **(95%CI)**
Lab-confirmed influenza, protocol-defined ILI [4]	15,991	301	1.9%	15,998	228	1.4%	24% (9.7 to 36)	0.46% (0.18 to 0.74)	4.6 (1.8 to 7.4)	219 (136 to 565)
Lab-confirmed influenza, modified CDC ILI definition [4]	15,991	121	0.8%	15,998	96	0.6%	21% (−4.6 to 40)	0.16% (−0.24 to 0.34)	1.6 (−2.4 to 3.4)	639 (298 to ∞)
Lab-confirmed influenza, any respiratory illness (all strains) [4]	15,991	387	2.4%	15,998	316	2.0%	18% (5.0 to 30)	0.44% (0.12 to 0.77)	4.4 (1.2 to 7.7)	225 (131 to 820)
Season 2012-13, predominantly antigenically matched B [4]	8749	65	0.7%	8737	45	0.5%	31% (−1.0 to 53)	0.23% (−0.0064 to 0.46)	2.3 (−0.064 to 4.6)	436 (22 to ∞)
Season 2012-13, predominantly antigenically unmatched H3N2 [4]	8749	243	2.8%	8737	204	2.3%	15.9% (−1.0 to 30)	0.44% (−0.025 to 0.91)	4.4 (−0.2 to 9.1)	222 (11 to ∞)
**B**	**SD-IIV**			**rIV**						
**Clinical endpoint**	**vaccinated**	**positive**	**event rate**	**vaccinated**	**positive**	**event rate**	**rVE (95%CI)**	**ΔRD %**	**ΔRD per 1000**	**ΔNNV**
Lab-confirmed influenza ILI [6]	4301	138	3.2%	4303	96	2.2%	30% (10 to 47)	0.98% (0.29 to 1.7)	9.8 (2.9 to 17)	102 (59 to 345)
Any H3N2 (predominantly antigenically mismatched) [6]	4301	110	2.66%	4303	71	1.65%	36% (14 to 53)	0.91% (0.30 to 1.5)	9.1 (3.0 to 15)	110 (66 to 332)
Any B (antigenically matched) [6]	4301	24	0.56%	4303	23	0.53%	4.2% (−72 to 46)	0.023% (−0.029 to 0.33)	0.23 (−0.29 to 3.3)	4256 (303 to ∞)
**C**	**SD-IIV**			**cIV**						
**Clinical endpoint**	**vaccinated**	**positive**	**event rate**	**vaccinated**	**positive**	**event rate**	**rVE (95%CI)**	**ΔRD %**	**ΔRD per 1000**	**ΔNNV**
Lab-confirmed influenza ILI [14]	3638	49	1.3%	3776	42	1.1%	17% (−24 to 45)	0.23% (−0.27 to 0.74)	2.3 (−2.7 to 7.4)	426 (13 to ∞)
H1N1 predominantly antigenically matched [14]	3638	8	0.22%	3776	5	0.13%	40% (−84 to 80)	0.087% (−0.0010 to 0.28)	0.87 (−0.010 to 2.8)	1143 (358 to ∞)
B predominantly antigenically mismatched [14]	3638	27	0.74%	3776	29	0.77%	−3.5% (−74 to 39)	−0.026% (−0.42 to 0.37)	−0.26 (−4.2 to 3.7)	<0
**D**	**SD-IIV**			**aIV**						
**Clinical endpoint**	**vaccinated**	**hospitalized**	**event rate**	**vaccinated**	**hospitalized**	**event rate**	**rVE (95%CI)**	**ΔRD %**	**ΔRD per 1000**	**ΔNNV**
Pneumonia and influenza hospitalization [13]	25,086	309	1.2%	24,926	242	0.9%	21% (6.7 to 33)	0.26% (0.08 to 0.44)	2.6 (0.8 to 4.4)	383 (225 to 1282)

## Data Availability

No new data were created or analyzed in this study. Data sharing is not applicable to this article.
